# Methylphenidate and the Response to Growth Hormone Treatment in Short Children Born Small for Gestational Age

**DOI:** 10.1371/journal.pone.0053164

**Published:** 2012-12-27

**Authors:** Judith S. Renes, Maria A. J. de Ridder, Petra E. Breukhoven, Annemieke J. Lem, Anita C. S. Hokken-Koelega

**Affiliations:** 1 Department of Pediatrics, Subdivision of Endocrinology, Erasmus MC/Sophia Children's Hospital, Rotterdam, The Netherlands; 2 Department of Biostatistics, Erasmus MC, Rotterdam, The Netherlands; 3 Dutch Growth Research Foundation, Rotterdam, The Netherlands; Tehran University of Medical Sciences, Iran (Islamic Republic of)

## Abstract

**Background:**

Growth hormone (GH) treatment has become a frequently applied growth promoting therapy in short children born small for gestational age (SGA). Children born SGA have a higher risk of developing attention deficit hyperactivity disorder (ADHD). Treatment of ADHD with methylphenidate (MP) has greatly increased in recent years, therefore more children are being treated with GH and MP simultaneously. Some studies have found an association between MP treatment and growth deceleration, but data are contradictory.

**Objective:**

To explore the effects of MP treatment on growth in GH-treated short SGA children

**Methods:**

Anthropometric measurements were performed in 78 GH-treated short SGA children (mean age 10.6 yr), 39 of whom were also treated with MP (SGA-GH/MP). The SGA-GH/MP group was compared to 39 SGA-GH treated subjects. They were matched for sex, age and height at start of GH, height SDS at start of MP treatment and target height SDS. Serum insulin-like growth factor-I (IGF-I) and IGF binding protein-3 (IGFBP-3) levels were yearly determined. Growth, serum IGF-I and IGFBP-3 levels during the first three years of treatment were analyzed using repeated measures regression analysis.

**Results:**

The SGA-GH/MP group had a lower height gain during the first 3 years than the SGA-GH subjects, only significant between 6 and 12 months of MP treatment. After 3 years of MP treatment, the height gain was 0.2 SDS (±0.1 SD) lower in the SGA-GH/MP group (P = 0.17). Adult height was not significantly different between the SGA-GH/MP and SGA-GH group (−1.9 SDS and −1.9 SDS respectively, P = 0.46). Moreover, during the first 3 years of MP treatment IGF-I and IGFBP-3 measurements were similar in both groups.

**Conclusion:**

MP has some negative effect on growth during the first years in short SGA children treated with GH, but adult height is not affected.

## Introduction

Short stature persists in approximately 10% of children born small for gestational age (SGA) [1]. Growth hormone (GH) treatment has become a frequently applied growth promoting therapy and results in significant catch-up growth [2]. Children born SGA have a higher risk of developing attention deficit hyperactivity disorder (ADHD) [3], [4]. ADHD is one of the most commonly encountered behavioral problems in childhood. Treatment of ADHD with stimulant drugs, such as methylphenidate (MP), has greatly increased in recent years [5], [6]. MP has been associated with growth deceleration, specifically during the first years of treatment [7], [8], [9], [10], [11], [12], [13], [14].

An increasing number of children are being treated with both GH and a stimulant drug [15]. Data regarding the effect of stimulant medication on growth in children treated with GH are limited. Children with idiopathic GH deficiency treated with GH are reported to have a significant smaller height gain if a stimulant drug is being used simultaneously [15], [16]. This suggests that simultaneous use of stimulant medication and GH might not be advised.

The cause of the decelerated growth during MP use has not yet been elucidated. One proposed mechanism is loss of appetite which leads to less weight gain [12]. Another possible cause may be the altered dopaminergic pathways in ADHD. These pathways are also involved in GH secretion and MP treatment decreases the re-uptake of dopamine and noradrenalin [17], [18], [19]. Both proposed mechanisms can lead to less GH secretion and lower serum levels of insulin-like growth factor I (IGF-I) and IGF-binding protein-3 (IGFBP-3), which both play a central role in postnatal growth.

We hypothesized that simultaneous use of GH and MP would negatively affect the growth response to GH therapy, because of lower serum IGF-I and IGFBP-3 levels. To determine the effect of MP on growth we ideally should have randomly assigned MP treatment to two groups of patients. However, since the effectiveness of MP has been established in various studies this was ethically not feasible [20]. In this paper we retrospectively report height and weight gain, and adult height in short SGA children treated with GH and MP and compare these data with matched GH-treated SGA subjects without MP. We also describe the effect of MP on serum IGF-I and IGFBP-3 levels.

## Patients and Methods

### Subjects

The study cohort consisted of 427 short children born SGA who participated in four GH trials. The inclusion criteria for these studies have previously been described [21], [22], [23], [24], [25]. In short, children were included according to the following criteria: 1) birth length and/or birth weight standard deviation score (SDS) below −2.0 for gestational age [26], 2) height SDS for calendar age (CA) below −2.0 according to Dutch standards at start of GH treatment [27], 3) height velocity SDS below zero to exclude children with spontaneous catch-up growth.

For the present study we included 78 subjects, 39 of them received MP for ≥6 months (SGA-GH/MP) and 39 GH treated children born SGA (SGA-GH) who had never been exposed to MP treatment. Each subject in the SGA-GH/MP treated group was individually matched to an SGA-GH subject based on sex, age and height at start of GH treatment, height SDS at start of MP treatment and target height (TH) SDS. The delta accepted for each parameter was 0.5 SDS. Of the 39 SGA-GH/MP subjects, 13 had been on MP treatment before the start of GH treatment. Study protocols were approved by the Medical Ethics Committee of all participating centers (Erasmus Medical Center, Admiraal de Ruyter Hospital, Canisius Hospital, Catharina Hospital, Free University Medical Center, Isala Clinics, Juliana Children's Hospital, Leiden University Medical Center, University Medical Center Groningen, University Medical Center Radboud, Rijnstate Hospital, Walcheren Hospital, Wilhelmina Children's Hospital, and Zaans Medisch Centrum) and written informed consent was obtained from all participants and/or their parents.

### Study design

During GH treatment, height, weight and Tanner stage were determined every 3 months as previously described [22]. Children were treated with biosynthetic growth hormone approximately 1 mg/m^2^/day which was administered once daily at bedtime. Body mass index (BMI) was calculated as weight divided by height squared (kg/m^2^). Target height (TH) was calculated as TH = [(maternal height+paternal height+13)/2+4.5] for boys and TH = [(maternal height+paternal height−13)/2+4.5] for girls. Height, BMI and TH were expressed in SDS, adjusting for sex and age according to Dutch reference data for children [27]. The onset of puberty was defined as breast development stage 2 according to Tanner for girls and testicular volume ≥4 ml for boys [28].

#### Biochemical measurements

Blood samples were taken at baseline and yearly during GH treatment. After centrifugation, all samples were frozen (−80°C) until assayed. RIA measurements of serum IGF-I and serum IGFBP-3 levels were performed in one laboratory as previously described [29]. Levels of IGF-I and IGFBP-3 were expressed as SDS, adjusting for age and gender, using reference values from healthy children of normal stature [30].

#### Statistical analysis

A paired-sample t-test was used to compare the SGA-GH/MP group with the matched controls in the SGA-GH group. The Wilcoxon signed-ranks test was used to compare age at start of puberty. Height gain SDS was analyzed from start of MP treatment over 3 years. If MP treatment had started before GH treatment we analyzed data from start of GH treatment. The longest consecutive period of MP treatment was used when analyzing the data. The changes over time were analyzed with repeated measures regression analysis to correct for multiple testing and missing data (≥1 yr use of MP in 36 subjects, ≥2 yrs n = 26 and ≥3 yrs n = 20). The effect of MP treatment was estimated separately for three time periods, the first 6 months, for 6 to 12 months and for the period after 12 months. Also, the effect for the period after stop of MP treatment was estimated. The analysis of height gain SDS was adjusted for sex, TH SDS, age at start of GH treatment, puberty, GH dose, gonadotropin releasing hormone analogue use (n = 8) and duration of GH treatment before start of MP treatment. The analysis of gain in BMI SDS was adjusted for the same variables except for TH SDS. Adult height (AH) was defined as the condition when height velocity dropped <0.5 cm during the previous 6 months. AH SDS was calculated based on Dutch reference data for age 21 years [27]. Corrected AH was calculated by subtracting the TH SDS from the AH SDS. Difference in AH SDS and corrected AH SDS within the matched pairs was analyzed with a repeated measures model correcting for age and height SDS at start of GH treatment, TH SDS, age at start of puberty and GH dose. IGF-I and IGFBP-3 SDS data were adjusted for duration of GH treatment before start of MP treatment, GH dose and puberty. A P-value<0.05 was considered significant. Analyses were performed using the statistical package SPSS for Windows (version 17.0; SPSS Inc., Chicago, IL). For repeated measures regression analysis SAS 8.2 (SAS Institute Inc., Cary, /nC, USA) was used.

## Results

### Baseline characteristics

Of the total study group, 39 subjects were treated with MP (∼10%). Clinical characteristics of the SGA-GH/MP and SGA-GH subjects are shown in Table 1. MP treatment was more common in boys than in girls (31 boys and 8 girls). There were no significant differences between the SGA-GH/MP and SGA-GH group. Mean (±SD) age at start of MP treatment was 10.6 (±2.2) yr and mean duration of MP treatment was 3.7 (±2.5) yr. The mean dose of MP was 28.2 (±13.4) mg/day.

**Table 1:** Patient characteristics.

	SGA-GH/MP	SGA-GH	P-value
Male/female	31/8	31/8	1.00
Age at start GH	8.7 (2.9)	8.7 (2.8)	0.93
Height SDS at start GH	−2.7 (0.9)	−2.8 (0.5)	0.60
Target Height SDS	−0.37 (0.7)	−0.40 (0.7)	0.72
Weight SDS at start MP	−2.1 (1.2)	−1.8 (1.2)	0.08
BMI SDS at start MP	−1.0 (1.0)	−0.7 (1.1)	0.18
Age at start puberty (yr), male	12.5 (10.2 to 14.7)	12.4 (10.0 to 12.4)	0.70
Age at start puberty (yr), female	11.0 (9.9 to 11.8)	10.9 (9.8 to 11.9)	0.89

Values are expressed as mean (SD). Age at start puberty is expressed as median (interquartile range). BMI = body mass index; GH = growth hormone; MP = methylphenidate; SDS = standard deviation score; SGA = small for gestational age.

### Growth

**Figure 1:**
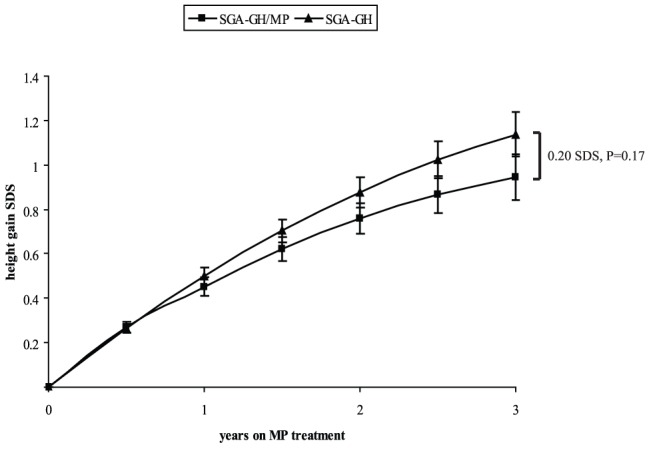
Height gain SDS during 3 years of MP treatment. Data are expressed as model estimates (standard error), ≥1 yr use of MP in 36 subjects, ≥2 yrs n = 26 and ≥3 yrs n = 20. GH = growth hormone; MP = methylphenidate; SGA = small for gestational age; SDS = standard deviation score.

Figure 1 shows the height gain SDS in the two groups. Height gain was not significantly different between the two groups during the first 6 months of MP treatment (0.005 SDS, P = 0.85), but between 6 and 12 months, the SGA-GH/MP group had a significantly smaller height gain SDS than the SGA-GH group (−0.05 SDS, P = 0.03). Between 1 and 3 years of MP treatment, the height gain SDS was also smaller in the SGA-GH/MP group but not significantly, with a difference of −0.04 SDS (P = 0.06) in every six months. After 3 years, the difference between the two groups amounted to 0.2 SDS (P = 0.17).

Adult height was reached in 25 of the 39 SGA-GH/MP subjects (64%) and in 28 of the 39 SGA-GH subjects (72%). Mean age at reaching AH was 17.3 (±1.2) yr for boys and 16.0 (±0.9) for girls in the SGA-GH/MP group and 16.9 (±1.2) yr for boys and 15.6 (±0.5) yr for girls in the SGA-GH group (boys P = 0.26 and girls P = 0.41). In the SGA-GH/MP group, the mean duration of MP treatment was 4.6 (±2.6) yr and of GH treatment was 8.1 (±2.5) yr. In the SGA-GH group the duration of GH treatment was 7.8 (±2.3) yr. Mean AH was −1.9 (±0.7) SDS in the SGA-GH/MP and −1.9 (±0.6) SDS in the SGA-GH group. AH between the groups was not significantly different (adjusted difference 0.07 SDS, P = 0.57). Corrected AH was −1.4 (±0.8) SDS in the SGA-GH/MP and −1.4 (±0.7) in the SGA-GH group, this was not significantly different (adjusted difference 0.09 SDS, P = 0.44).

At start of MP treatment, the BMI SDS was −1.0 (±1.0) SDS in the SGA-GH/MP group and −0.7 (±1.1) SDS in the SGA-GH subjects. During the first 6 months of MP treatment, the gain in BMI SDS was not significantly different between the two groups (−0.09 SDS, P = 0.13). However, between 6 and 12 months the SGA-GH/MP group had a significantly larger gain in BMI SDS than the SGA-GH group (0.13 SDS, P = 0.02). Between 1 and 3 years, the six-monthly BMI gain SDS was not significantly different between the two groups (0.02 SDS, P = 0.52).

After cessation of MP treatment, there was no acceleration of growth after six months (height gain SDS 0.03 SDS, P = 0.36) compared to the controls. There was also no acceleration in BMI SDS gain, six months after discontinuation of MP (0.02 SDS, P = 0.74).

### IGF-I and IGFBP-3 levels

**Figure 2:**
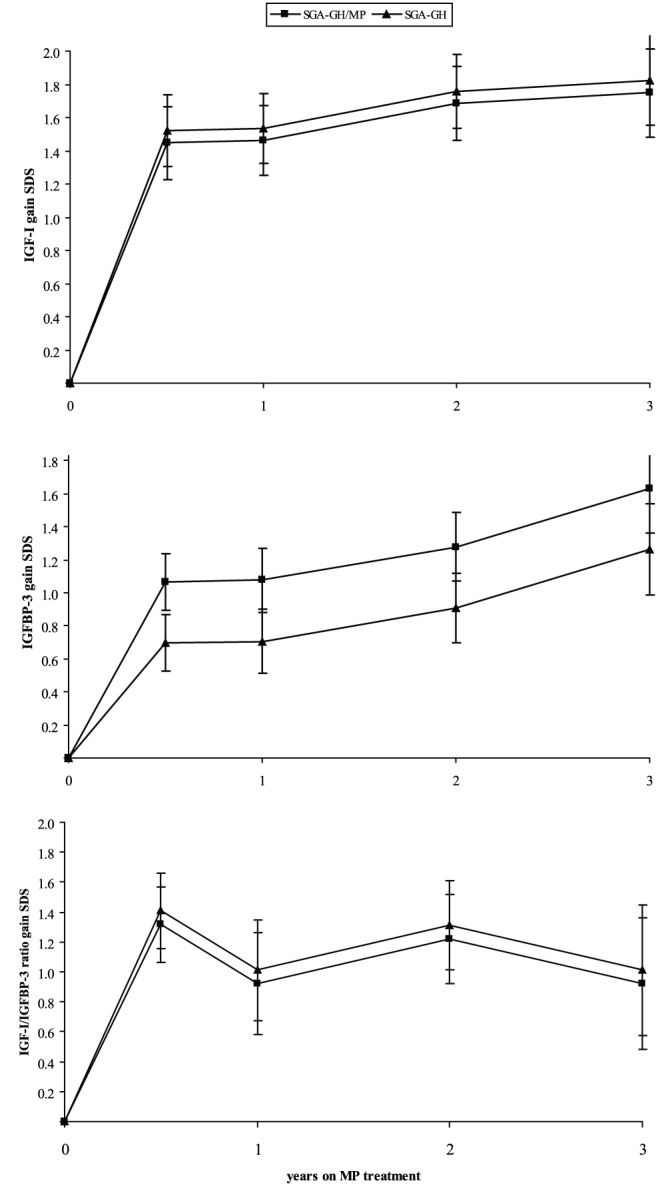
IGF-I, IGFBP-3 and IGF-I/IGFBP-3 ratio gain SDS values during 3 years of MP treatment. Data are expressed as model estimates (standard error). IGF-I = insulin-like growth factor; IGFBP-3 = insulin-like growth factor binding protein-3; MP = methylphenidate; SGA = small for gestational age; SDS = standard deviation score.

Serum IGF-I and IGFBP-3 SDS levels are shown in Figure 2. The gain in IGF-I SDS was not significantly different between the SGA-GH/MP and SGA-GH group during three years of MP treatment (−0.07 SDS, P = 0.75). The gain in serum IGFBP-3 SDS was higher in the SGA-GH/MP group, but not significantly so (0.37 SDS, P = 0.06). The IGF-I/IGFBP-3 ratio SDS was also not significantly different between the SGA-GH/MP and SGA-GH group (−0.09 SDS, P = 0.76).

## Discussion

In this study we investigated the effect of methylphenidate (MP) treatment on growth in short children born SGA treated with GH. We analyzed growth during the first 3 years of MP in addition to GH treatment and also adult height. We showed that the 3-year height gain was 0.2 SDS smaller in children treated with MP than in non-MP-treated children, but not significant. Reassuringly, there was no difference in adult height between the SGA-GH/MP group and the SGA-GH group. The gain in BMI SDS was not negatively affected by MP treatment and IGF-I and IGFBP-3 levels were also not significantly different between the two groups.

ADHD is a disorder of hyperactivity, inattention and impulsivity that affects approximately 5% of children in the Netherlands [6]. The most frequently prescribed drugs in the treatment of ADHD are stimulant drugs, such as MP [5], [6]. Since the use of stimulant drugs has increased substantially in recent years more children are being treated with GH and MP simultaneously [5], [6], [15]. In our cohort of short SGA children, approximately 10% were being treated with MP, which is considerably higher than the reported 5%, indicating that ADHD seems to occur more frequently in children born SGA. This is in line with data reported by van der Reijden-Lakeman and Groen-Blokhuis et al [4], [31].

Simultaneous use of MP during GH treatment has been associated with growth deceleration, specifically during the first years of treatment [15], [16]. We showed that after 3 years of GH treatment, the difference between the SGA-GH/MP and SGA-GH group was 0.2 SDS. These results are in line with Rao et al., who reported a negative effect on height gain of 0.17 SDS during 3 years of MP treatment [16]. It demonstrates that there is a small but distinct risk of diminished growth during the first years of MP treatment in GH-treated short SGA children.

Data on adult height are very limited. To our knowledge, there are no reports on adult height in subjects treated with both GH and MP. Our study shows no significant difference in adult height SDS between the SGA-GH/MP and SGA-GH group (0.07 SDS, p = 0.46) which suggests that the initial loss of height gain has no long-term adverse effect on adult height. Earlier reports investigating adult height in children treated with only MP did not find any effects either, despite significant effects on height and weight during the first years of treatment [32], [33], [34], [35]. A possible explanation might be catch-up growth after cessation of MP treatment. However, we found no increase in height gain SDS 6 months after discontinuation of MP (0.03 SDS, P = 0.36), despite reports that cessation of MP treatment results in catch-up growth [36], [37], [38].

The mechanism by which MP affects growth is not completely understood. Decreased appetite leading to reduced weight gain has been proposed as a cause of growth reduction [12]. We did not find that MP negatively influenced gain in BMI SDS during three years of treatment. In fact, during the second half of the first year of MP treatment gain in BMI SDS was higher in the SGA-GH/MP group. This might be explained by the low weight SDS at the start of MP treatment (−2.1 SDS). Parents are perhaps being made more aware of reduced appetite as possible side effect of MP and tend to “overfeed” their children to prevent a reduction in weight. Furthermore, the negative effect of MP on weight gain appears to be more pronounced in children who are overweight, which is not the case in short SGA children [10], [39].

Another cause of the decelerated growth may be that MP has a negative influence on the re-uptake of dopamine, a hormone that is involved in the regulation of GH secretion. The effects of GH are mediated by IGF-I. Although gain in serum IGF-I levels was lower in the SGA-GH/MP group, this difference was not significant. Gain in serum IGFBP-3 levels was higher in the SGA-GH/MP group, but again not significantly so. The lower serum IGF-I and higher serum IGFBP-3 levels could result in reduced free IGF-I levels. However, the gain in IGF-I/IGFBP-3 ratio SDS was not significantly lower in the SGA-GH/MP group. Other studies on the effect of MP on GH, serum IGF-I and IGFBP-3 levels are contradictory. Bereket et al showed that MP caused a transient decrease in IGF-I and IGFBP-3 levels [19]. On the other hand a study by Toren et al. found no difference in GH, GH binding protein and IGF-I levels [40]. Our results suggest that the cause of growth deceleration does not seem to be the result of significantly lower IGF-I and IGFBP-3 levels.

In this study we measured height and weight every 3 months enabling us to accurately assess growth patterns. In this study thirteen subjects had been treated with MP before the start of GH. Since the negative effects on growth are most pronounced during the first years of MP treatment, it is possible that we have underestimated the decrease in height gain. Furthermore, although our study followed the growth of short SGA children treated with both GH and MP, we did not follow a stimulant-untreated ADHD control group. Ideally the start of stimulant medication should be randomized. However, since the efficacy of MP treatment has been well established such a design was considered unethical [20].

In conclusion, physicians should be aware that height gain can be somewhat reduced during the first years of MP treatment in children treated with GH. It is however reassuring that adult height does not seem to be affected. Growth deceleration during the first years of MP treatment does not seem to be mediated through a reduced BMI gain or a reduction in IGF-I and IGFBP-3 levels.
